# Ionic Liquid-Catalyzed Green Protocol for Multi-Component Synthesis of Dihydropyrano[2,3-*c*]pyrazoles as Potential Anticancer Scaffolds

**DOI:** 10.3390/molecules22101628

**Published:** 2017-09-28

**Authors:** Urja D. Nimbalkar, Julio A. Seijas, Maria Pilar Vazquez-Tato, Manoj G. Damale, Jaiprakash N. Sangshetti, Anna Pratima G. Nikalje

**Affiliations:** 1 Maulana Azad Post Graduate and Research Centre, Dr. Rafiq Zakaria Campus, Rauza Baug, Aurangabad 431001, India; urjasatish@gmail.com; 2 Departamento de Química Orgánica, Facultad de Ciencias, Universidad of Santiago de Compostela, Alfonso X el Sabio, 27002 Lugo, Spain; julioa.seijas@usc.es (J.A.S.); pilar.vazquez.tato@usc.es (M.P.V.-T.); 3 Shreeyash Institute of Pharmaceutical Education and Research, Aurangabad 431010, India; pharmalink1985@gmail.com; 4 Y.B. Chavan College of Pharmacy, Dr. Rafiq Zakaria Campus, Rauza Baug, Aurangabad 431001, India; jnsangshetti@rediffmail.com

**Keywords:** ionic liquid, multi-component synthesis, dihydropyrano[2,3-*c*]pyrazoles, anticancer activity, ADMET prediction, molecular docking study

## Abstract

A series of 6-amino-4-substituted-3-methyl-2,4-dihydropyrano[2,3-*c*]pyrazole-5-carbonitriles **5a**–**j** were synthesized via one-pot, four-component condensation reactions of aryl aldehydes **1a**–**j**, propanedinitrile (**2**), hydrazine hydrate (**3**) and ethyl acetoacetate (**4**) under solvent-free conditions. We report herein the use of the Brønsted acid ionic liquid (BAIL) triethylammonium hydrogen sulphate [Et_3_NH][HSO_4_] as catalyst for this multi-component synthesis. Compared with the available reaction methodology, this new method has consistent advantages, including excellent yields, a short reaction time, mild reaction conditions and catalyst reusability. Selected synthesized derivatives were evaluated for in vitro anticancer activity against four human cancer cell lines viz. melanoma cancer cell line (SK-MEL-2), breast cancer cell line(MDA-MB-231), leukemia cancer cell line (K-562) and cervical cancer cell line (HeLa). Compounds **5b**, **5d**, **5g**, **5h** and **5j** exhibited promising anticancer activity against all selected human cancer cell lines, except HeLa. Molecular docking studies also confirmed **5b** and **5d** as good lead molecules. An in silico ADMET study of the synthesized anticancer agents indicated good oral drug-like behavior and non-toxic nature.

## 1. Introduction

**Figure 1:**
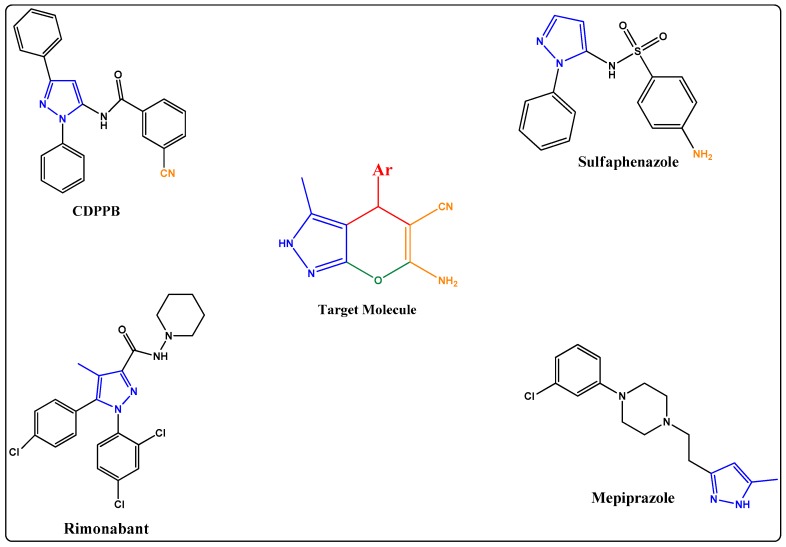
Design protocol for the target molecules.

According to data from the World Health Organization (WHO), more than 13% of all deaths worldwide are directly caused by cancer every year, making cancer one of the most public-threatening diseases [1,2]. As per the annual report of the U.S. NIH on the status of cancer from 1975–2014, though cancer death rates continue to decrease in the United States, even then, progress in reducing death rates and improving survival is limited for several cancer types, underscoring the need for intensified efforts to discover new strategies for its prevention, early detection and treatment and to apply proven preventive measures broadly and equitably. This necessitates continued cancer drug discovery efforts [3]. Chemotherapy is still the main treatment for cancer and existing chemotherapeutic agents are accompanied by various detrimental side effects. This clearly motivates the crucial need to design novel chemotherapeutic agents with more compelling antitumor activities and reduced side effects. Pyrazoles are an important class of heterocycles exhibiting numerous biological activities and a widely studied scaffold for their anticancer activity, especially on the human cancer cell lines SK-MEL-2, MDA-MB-231, K-562 and HeLa [4,5,6,7,8,9,10]. It has been proven by many studies that pyranopyrazoles obtained by coupling pyran rings with pyrazoles have importance for their potential to show numerous biological activities [11,12,13,14], including anticancer activity [15,16,17,18,19]. Hadi Adibi et al. [20] reported the in vitro anticancer activity of pyaranopyrazoles against human liver carcinoma (HepG2), human mouth carcinoma (KB), human colon adenocarcinoma (SW48), and human lung carcinoma (A549) cell lines. Mona Kamel [21] reported in vitro anticancer activity against human gastric cancer (NUGC), human colon cancer (DLD1), human liver cancer (HA22T and HEPG2), nasopharyngeal carcinoma (HONE1), and human breast cancer (MCF). However, our literature survey indicated that the potential in vitro anticancer activity of pyranopyazoles against SK-MEL-2, MDA-MB-231, K-562 and HeLa had not yet explored for, therefore, the above cell lines were selected for anticancer evaluation. In continuation of our search for green synthesis methods for novel heterocycles as anticancer agents [22,23,24] we report herein the ionic liquid-assisted multi-component synthesis of ten dihydropyrano[2,3-*c*]pyrazoles derivatives. The research protocol was designed taking into consideration the existing anticancer drugs containing a pyrazole ring as an important pharmacophore. In this research work the pyrazole ring was coupled with 6-amino-2*H*-pyran-5-carbonitrile. Thus, the designed structure contains coupled important pharmacophores like pyrazoles, pyrans, amino groups and nitrile groups. These coupled synthesized derivatives have exhibited excellent in vitro anticancer activity. The design protocol is given in Figure 1.

Synthesis of dihydropyrano[2,3-*c*]pyrazoles can be achieved by various methodologies. Several catalysts including trimethylamine [25,26,27], piperazine [28], piperidine [29], *N*-methylmorpholine [30], heteropolyacids [31], glycine [32], per-6-amino-β-cyclodextrin [33], Mg/Al hydrotalcite [34], nanosized magnesium oxide [35], L-proline [36], γ-alumina [37], sodium benzoate [38], Amberlyst A21 [39] and CTACl [40], [Bmim]BF_4_ [41], PS-PTSA [42], urea [43] and ethanol and water (1:1) [44] are reported in the literature. Ionic liquids have gained importance as green solvent for organic transformations [45,46,47] due to their special properties, including good solvating capability, negligible vapor pressure, non-inflammable, ease of recyclability, controlled miscibility and high thermal stability [48,49]. Use of the Brønsted acid ionic liquid (BAIL) triethylammonium hydrogen sulphate ([Et_3_NH][HSO_4_] [50,51,52,53,54,55,56,57,58,59,60] as a catalyst should be advantageous due to its non-toxic nature, low cost, easy preparation from readily available starting materials, stability in water and air, easy separation and reusability [61,62].

In the area of synthetic organic chemistry, multi-component reactions between three or more components in one pot to give new ‘drug-like’ molecules with the essential parts of all the initial reactants represent a time honored efficient and prominent tool. Multi-component reactions offer significant advantages such as variety of convergent synthesis of complex organic compounds, facile mechanism, atom economy, low cost, shorter reaction and work-up time, easy purification processes and minimum wastage that all contribute to an environmentally friendly method of synthesis of simple and complex building blocks [63,64]. In continuation to our earlier efforts [65] studying ionic liquid-assisted synthesis, herein we report multi-component reactions for the synthesis of 6-amino-4-substituted-3-methyl-2,4-dihydropyrano[2,3-*c*]pyrazole-5-carbonitriles **5a**–**j** giving excellent yields using the ionic liquid [Et_3_NH][HSO_4_] as a catalyst as well as the reaction medium. Numerous classes of heterocycles such as pyrazoles, chromones, indolines, stilbene derivatives have exhibited anticancer activity and their potential was evaluated against human cancer cell lines viz. melanoma (SK-MEL-2), breast cancer (MDA-MB-231), leukemia (K-562) and cervical cancer (HeLa) cell lines. Their molecular basis of inhibition was determined by docking against tubulin in place of co-crystallized ligand molecule. In the present study, the synthesized compounds were evaluated for in vitro anticancer activity by the SRB assay against the four aforementioned human cancer cell lines using Adriamycin (ADR) as a standard reference drug and based on this assumption the synthesized compounds were docked in the active site of tubulin to find a possible mode of inhibition.

## 2. Results

### 2.1. Chemistry

**Scheme 1:**
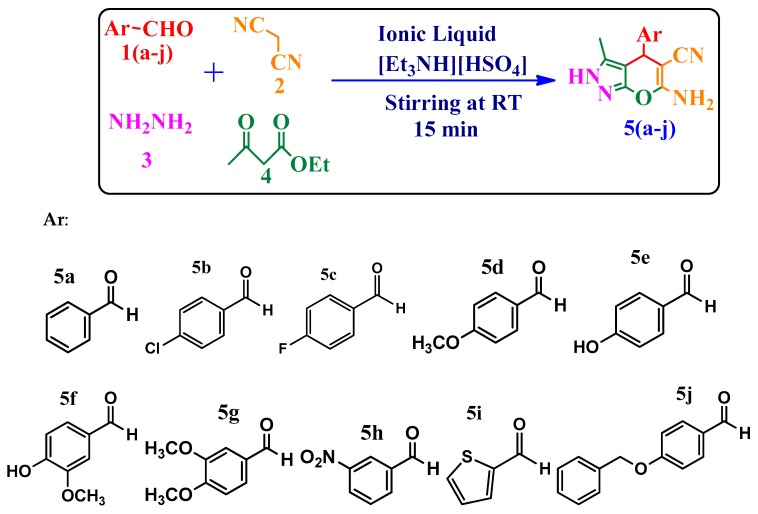
One pot, four component synthesis of 6-amino-4-substituted-3-methyl-2,4-dihydropyrano[2,3-*c*]pyrazole-5-carbonitriles **5a**–**j**.

All the final 6-amino-4-substituted-3-methyl-2,4-dihydropyrano[2,3-*c*]pyrazole-5-carbonitrile compounds **5a**–**j** were synthesized following the procedure depicted in Scheme 1. All the reactants were added in one pot in equimolar ratios starting with a substituted aromatic aldehydes **1a**–**j** (1 mmol), propanedinitrile (**2**, 1 mmol), hydrazine hydrate (**3**, 1 mmol) and ethylacetoacetate (**4**, 1 mmol) in 20 mol % ionicl iquid [Et_3_NH][HSO_4_] as a reaction catalyst as well as the reaction medium and the mixture was stirred at room temperature for 15 min. After completion of the reaction, which was followed by TLC analysis, the reaction was quenched with crushed ice and the mixture extracted with ethyl acetate. The obtained products **5a**–**j** were recrystallized from ethanol and the corresponding target compounds **5a**–**j** were obtained in good yields, as mentioned in Table S4, provided in the Supplementary File. The structures of the final products were confirmed on the basis of their respective analytical and spectral data. Spectra of the synthesized compounds are provided in the Supplementary Data, File S: 1. The drawn structures of each of the final compounds **5a**–**j** along with their IUPAC names are given in Supplementary Data, File S: 5.

Taking into consideration the significance of green chemistry, efforts were directed towards the use of all green reaction media. The model reaction was performed separately in various green solvents viz. (1) polyethylene glycol, (2) deep eutectic solvent of choline chloride: urea [66], (3) the ionic liquid *N*-methyl pyridiniumtosylate [67] and (4) the ionic liquid triethylammonium hydrogen sulfate [Et_3_NH][HSO_4_] [68]. 

For the selection of reaction medium and optimization of the reaction temperature and time of completion of the reactions, the model reaction was carried out in four sets with four green reaction media containing four components in one pot using *p*-chlorobenzaldehyde (**1b**) propanedinitrile (**2**), hydrazine hydrate (**3**) and ethylacetoacetate (**4**) as the reactants. The temperature required for each set of reactions, completion time and percent yield of product **5b** varies in each set as indicated in Table 1. The ionic liquid triethylammonium hydrogen sulphate ([Et_3_NH][HSO_4_]) was found to be the most suitable medium for the synthesis. Its use facilitated the reaction without the use of hazardous solvents, higher temperature, convetional refluxing method and gave the maximum yield amongst the tested solvents as shown in the Table 1.

Without the use of solvent/catalyst, noproduct formation was observed. Therefore, the catalyst [Et_3_NH][HSO_4_], was added in a model reaction in gradually increasing amounts to determine the optimum concentration of the selected ionic liquid.

With different concentrations of [Et_3_NH][HSO_4_], viz., 0, 5, 10, 15, 20 and 25 mol % 6-amino-4-(4-chlorophenyl)-3-methyl-2,4-dihydropyrano[2,3-*c*]pyrazole-5-carbonitrile **5b** was obtained in 0%, 50%, 65%, 70%, 94% and 85% yield, respectively, as shown in Table 2. A concentration of 20 mol % of [Et_3_NH][HSO_4_] was thus optimal for the reaction and hence it is selected for the synthesis of further derivatives of the series **5a**–**j**.

After completion of the reaction, the reusability of the residual ionic liquid was determined by washing it with diethyl ether and drying under vacuum at 60 °C. The recovered ionic liquid could be used for at least four times without much loss of catalytic activity, as shown in Table 3.

Thus, the multi-component, one pot condensations of 6-amino-4-(substituted phenyl)-3-methyl-2,4-dihydropyrano[2,3-*c*]pyrazole-5-carbonitrile were successfully completed using 20 mol % of the Brønsted acid ionic liquid [Et_3_NH][HSO_4_], as a catalyst and also as a green medium at room temperature for 15 min with excellent yield.

**Figure 2:**
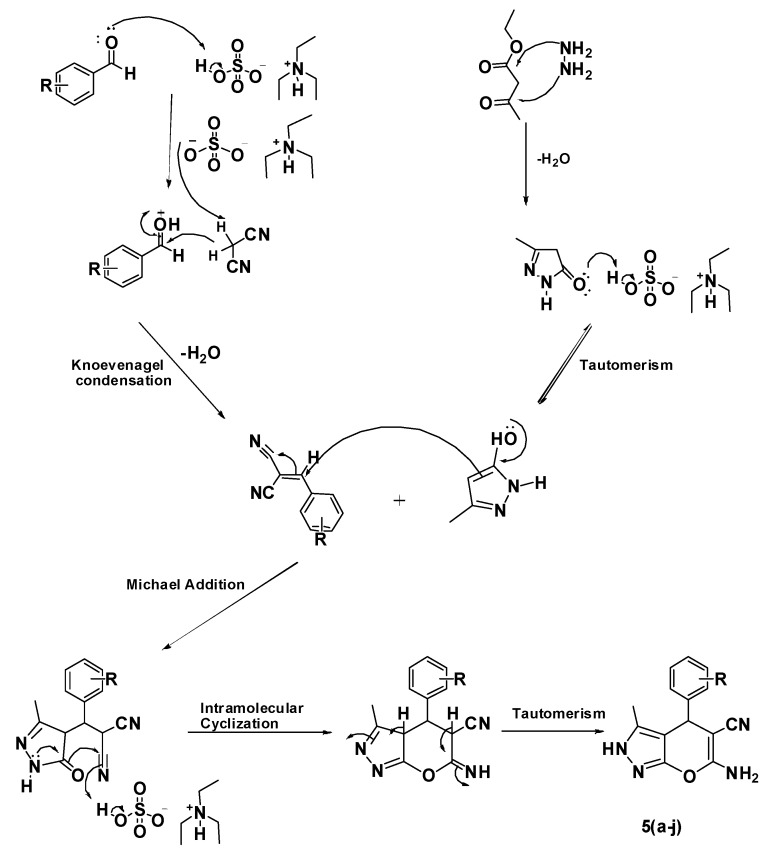
Plausible mechanism for the synthesis of 2,4-dihydropyrano[2,3-*c*]pyrazole.

A plausible mechanism for the ionic liquid-catalyzed synthesis of the 2,4-dihydropyrano[2,3-*c*]pyrazoles is shown in Figure 2. The Brønsted acid ionic liquid triethylammonium hydrogen sulphate increases the electrophilicity of the carbonyl carbon of the aromatic aldehydes **1a**–**j**. The conjugate base of the ionic liquid abstracts a proton from the active methylene group of propanedinitrile **(2)** to form a carbanion.

This carbanion attacks the electrophilic carbonyl carbon of the aromatic aldehydes in a Knoevenagel condensation reaction to give benzylidenepropanedinitrile as an intermediate. Hydrazine hydrate (**3**) and ethylacetoacetate (**4**) undergoes an acid-catalyzed condensation to give a pyrazolone. Michael addition between the pyrazolone and benzylidenepropanedinitrile followed by an acid-catalyzed intramolecular cyclization gives the final 6-amino-4-(substituted phenyl)-3-methyl-2,4-dihydropyrano[2,3-*c*]pyrazole-5-carbonitrile products **5a**–**j**.

### 2.2. In Vitro Anticancer Activity

The in vitro anticancer activities of the 6-amino-4-(substituted phenyl)-3-methyl-2,4-dihydro-pyrano[2,3-*c*]pyrazole-5-carbonitrile derivatives were examined against four human cancer cell lines, representing tumors of different origin. Each cell line melanoma (SK-MEL-2), breast (MDA-MB-231), leukemia (K-562) and cervix (HeLa) was incubated in culture medium with varying concentrations of the compounds and the effect was measured by a SRB assay, using the anticancer drug ADR as positive control. The GI_50_ values obtained with the selected cell lines are summarized in Table 4. The synthesized derivatives exhibited excellent anticancer activity against the tested cell lines except for the HeLa cell line. Compound **5b** has shown most potent anticancer activity, followed by **5d**.

In the in vitro anticancer activity, on the basis of results of growth inhibition curve plotted against molar concentration of synthesized derivatives for each selected cancer cell lines and the images of in vitro anticancer activity data are provided in Supplementary Data, File S2: In vitro anticancer activity images and File S3: Graphical representation of growth inhibition curve.

### 2.3. Molecular Docking

In order to explore the binding affinity, binding mode and molecular interactions of the synthesized derivatives a molecular docking study was carried out. Tubulin α/β heterodimer represents an important drug target in breast cancer [69]. Tubulin heterodimers of α- and β-tubulin (50 kDa each in size) are the basic structural components of microtubules which are hollow tubes of approximately 25 nm in diameter. Microtubules are cytoskeletal polymers involved in many cell functions such as mitosis, organization of intracellular structure and intracellular transport, as well as ciliary and flagellar motility. α,β dimer in relation to the polarity of the microtubule lattice displays β-tubulin monomer at the plus end and the α-tubulin is exposed at the minus end. In humans, there are six α-tubulin isotypes of and seven β-tubulin isotypes, and the level of expression of each isotype varies in different tissues and cells [70,71,72]. Of course, tubulin-binding drugs have different affinities for different isotypes, which affects the overall efficacy in different cancers. There are many chemically diverse compounds that bind to the tubulin–microtubule system. Tubulin-binding agents are potent mitotic poisons [73,74]. To perform our molecular docking study a three dimensional X-ray crystal structure of tubulin complex with colchicine (**PDB ID: 1SA0**) and a stathmin-like domain was used [75]. The docking study was carried out using the Surflex-Dock module of the Sybyl 2.1.1 package following the standard procedure.

To present the details of the docking scores shown in Table 4 the following terms are used: (a) total score as a total docking score; (b) crash score as degree of inappropriate penetration by the ligand into the protein and of interpenetration between ligand atoms that are separated by rotatable bonds of compounds; (c) polar score gives an idea about the contribution of the polar non-hydrogen bonding interactions to the total score.

All the synthesized compounds that were evaluated for anticancer activity on the cell lines have shown very good total docking scores, indicating the binding interactions in the active site and affinity of compounds and the target protein. The compounds that showed high cell toxicity indicated by their GI_50_ values on the cell lines all had very high total docking scores, polar scores and low crash scores indicating non-covalent interactions such as hydrogen bond interactions and π interactions [76].

**Figure 3:**
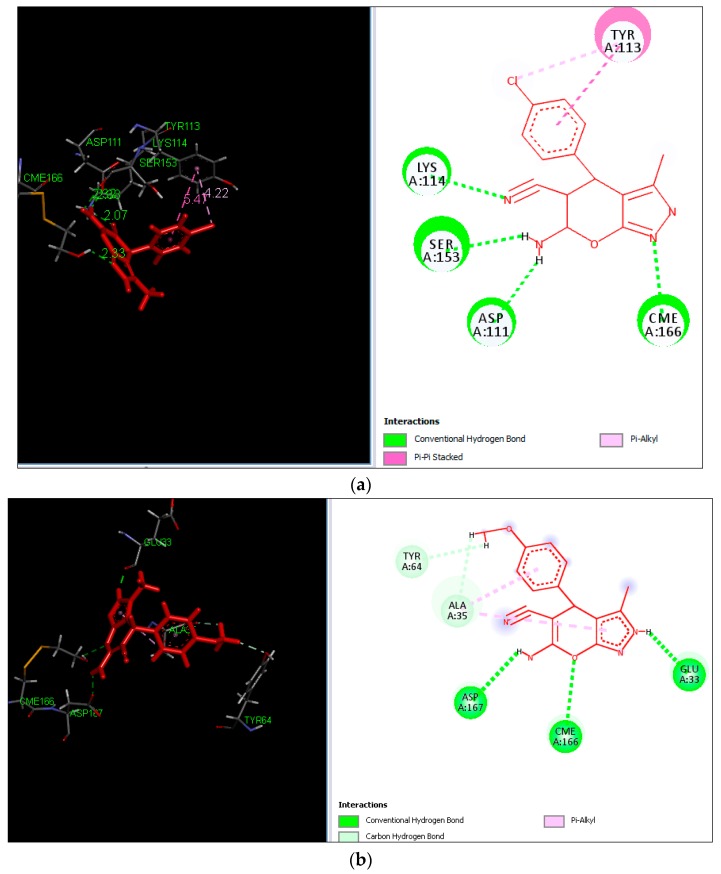
Binding poses and molecular interactions of (**a**) compound **5b** and (**b**) compound **5d** into the active site of tubulin.

The detailed analysis of binding affinity (−logki) values and the molecular interactions of dihydropyrano[2,3-*c*]pyrazole derivatives such as **5b** (6.4039) and **5d** (5.4734) suggests that they are the most active among all the synthesized derivatives and compared with the reference co-crystallized ligand CL2. The compounds **5g**, **5h** and **5j** had weak potential cytotoxicity indicated by low total docking scores, polar scores and high crash scores. The most active dihydropyrano[2,3-*c*]pyrazole derivatives **5b** and **5d** have shown efficient binding mode and penetration of the active site cavity by forming hydrogen bond interactions with active site residues such as ASP111, TYR113, LYS114,SER153 and CME166 as shown in Figure 3a,b. The most active derivative **5b** interacts with active site residue ASP111 and SER153 forming hydrogen bond interactions with the -NH_2_ groups of the dihydropyrano moiety with a distance of 2.03 Å and 2.77 Å, respectively. The amino acids LYS114 and CME166 formed hydrogen bonds with the cyano group and pyrazole nitrogen with distances of 2.33 Å and 2.33 Å, respectively. The hydrophobic amino acid TYR113 formed π interactions, π-π stacked and π-alkyl interactions with the chlorosubstituent and the aryl ring. The second most active dihydropyrano[2,3-*c*]pyrazole derivative **5d** (5.4734) formed hydrogen bonds and π interactions with amino acids such as GLU33, ALA35, TYR64 CME166 and ASP167. On the basis of GI_50_ activity data and molecular docking analysis, it was found that the dihydropyrano[2,3-*c*]pyrazole derivatives **5b** and **5d** have the potential to inhibit enzyme tubulin α/β.

### 2.4. Prediction of ADMET Properties

An in silico ADMET study was performed to evaluate the pharmacokinetics and safety potential of the synthesized dihydropyrano[2,3-*c*]pyrazole derivative **5a**–**j**. To predict the ADMET properties the ADMET predictor FAFDrugs2 which runs on Linux OS was used. This tool is freely available and used for in silico ADMET filtering [77,78]. In particular, we have calculated the compliance of synthesized compounds to Lipinski’s Rule of Five [79].

This approach has been widely used as a filter for lead molecules that could be further developed for drug design programs. We have assessed parameters like molecular percent absorption (%ABS < 100), weight (MW < 500), partition coefficient (logP < 5), number of rotatable bonds (<10), number of rigid bonds (<25) and ratio of H/C (<1). All the above mentioned parameters indicate the oral bio-availability and good intestinal absorption [80]. The values obtained are listed in Table 5.

Topological polar surface area (TPSA) i.e., the surface of any polar atoms, and molecular weight are the descriptors that are correlated with passive molecular transport through membranes that allows prediction of the route of transport of drugs through the barrier membranes in the intestine and blood-brain barrier (BBB). The percentage of absorption (% ABS) was calculated using TPSA by using the formula % ABS = 109 − (0.345 × TPSA) [81]. All the synthesized compounds exhibited a very good % ABS, ranging from 62.93% to 78.64%. The values of partition coefficient (logP < 5), number of rotatable bonds (<10), number of rigid bonds (<25) and ratio of H/C (<1) determine the absorption performance through the lipophilic phospholipid membranes and toxicity. None of the dihydropyrano[2,3-*c*]pyrazole derivatives **5a–j** violated Lipinski’s Rule of Five. All the dihydropyrano[2,3-*c*]pyrazole derivatives thus have the potential to be developed as an orally active drug candidate and could be potentially active anticancer drug candidates against the tested SK-MEL-2 melanoma cancer cell line, K-562 leukemia cell line and MAD-MB-321 breast cancer cell line.

## 3. Discussion

The results indicated that out of the ten synthesized derivatives compound **5b**, **5d**, **5h**, **5g** and **5j** exhibited significant cancer cell growth inhibition against the SK-MEL-2, MDA-MB-231 and K-562 cancer cell lines. Compound **5b**, which has chlorine as an electron-donating group at the *para* position of the phenyl ring exhibited excellent in vitro anticancer activity against the SK-MEL-2, MDA-MB-231 and K-562 cell line with GI_50_ concentrations of <0.1 µM, 0.74 µM and 11.20 µM, respectively. Compound **5d** bearing a strong electron-donating and lipophilic methoxy group at the *para* position of the phenyl ring also exhibited excellent anticancer activity against the melanoma, leukemia and breast cancer cell lines, with GI_50_ concentrations of <0.1 µM, 6.41 µM and 25.76 µM, respectively. Compound **5g** which bears two lipophilic and electron-donating methoxy groups at the *meta* and *para* positions of the phenyl ring, however showed moderate activity against the breast cancer, melanoma and leukemia cell lines, with GI_50_ concentrations of 2.02 µM, 2.92 µM and 40.73 µM, respectively, which may be due to the steric hindrance of the large substituent. Compound **5h** having a strong electron-withdrawing nitro group at the *meta* position of the phenyl ring also exhibited good anticancer activity against the three cell lines, with GI_50_ concentrations of 0.12 µM, 4.57 µM and 49.84 µM, respectively. Compound **5j** having benzyloxy group at para position exhibited excellent anticancer activity against cancer cell line SK-MEL-2 with <0.1 µM and moderate activity against K-562 and MDA-MB-231cell lines with concentration of 9.47 µM and 66.65 µM, respectively. No anticancer activity was observed against the HeLa cell line for any of the synthesized compounds.

According to the molecular docking study, the binding affinity (−logki) values and molecular interactions of the dihydropyrano[2,3-*c*]pyrazole derivatives **5b** and **5d** are 6.4039 and 5.4734 respectively, suggesting that they are the most active anticancer derivatives amongst all the synthesized derivatives when compared with the reference co-crystallized ligand CL2.

On the basis of in vitro anticancer activity and molecular docking as well as in silico ADMET study, it can be concluded that 6-amino-4-(substituted phenyl)-3-methyl-2,4-dihydropyrano[2,3-*c*] pyrazole-5-carbonitriles have potential to be developed as anticancer drug and can act as an excellent scaffold for lead optimization.

## 4. Materials and Methods

### 4.1. General Information

All the chemicals used for the synthesis were procured from Merck (Mumbai, India), Sigma (Mumbai, India), HiMedia (Mumbai, India) or Qualigens (Mumbai, India) and used without further purification. The progress of each reaction was monitored by ascending thin layer chromatography (TLC) using pre-coated silica gel F254 Alumina TLC Plates (Merck) and the spots were visualized with UV light and iodine vapor. Elemental analyses (C, H, and N) were done with a Flashea 112 analyzer (Shimadzu, Mumbai, India) and all analyses were consistent (within 0.4%) with theoretical values. IR spectra were recorded on a PS 4000 FTIR instrument (Jasco, Tokyo, Japan) using KBr pellets. ^1^H-(400 MHz) and ^13^C-NMR (100 MHz) spectra were recorded in DMSO-*d*_6_ on an Avance 400 NMR spectrometer (Bruker, Billerica, MA, USA) fitted with an Aspect 3000 computer and all the chemical shifts (δ ppm) were referred to internal TMS for ^1^H and the solvent signal for ^13^C-NMR. ^1^H-NMR data are reported in the order of chemical shift, multiplicity (s, singlet; d, doublet; t, triplet; q, quartet; br, broad; br s, broad singlet; m, multiplet and/or multiple resonance), number of protons. A Micro TOF-Q-II (Bruker Daltonics, Billerica, MA, USA) with electron spray ionization (ESI) was used to obtain the HRMS data.

### 4.2. Synthesis of [Et_3_NH][HSO_4_]

Sulphuric acid (98% solution of 0.02 mol) in water was dropped into triethylamine (0.02 mol) with stirring at 60 °C for 1 h. After the addition was complete, the reaction mixture was stirred for another 1 h at 70 °C. The water was removed by heating the residue at 80–90 °C under high vacuum until the weight of the residue remained constant.

### 4.3. Synthesis of 6-Amino-4-(substituted phenyl)-3-methyl-2,4-dihydropyrano[2,3-*c*]pyrazole-5-carbonitriles

A mixture of substituted aromatic benzaldehyde **1a**–**j** (1 mmol), malononitrile (**2**, 1 mmol), hydrazine hydrate (**3**, 1 mmol), and ethyl acetoacetate (**4**, 1 mmol) was added to 20 mol % [Et_3_NH][HSO_4_] and then the reaction mixture was stirred at room temperature. Progress of the reaction was monitored by TLC (ethyl acetate:*n*-hexane 1:9). After 15 min of stirring, the reaction was quenched with crushed ice and the mixture was extracted with ethyl acetate. The obtained crude compounds were recrystallized from ethanol. An important feature of this method is that all the synthesized derivatives were obtained in excellent yields.

*6-Amino-3-methyl-4-phenyl-2,4-dihydropyrano[2,3-c]pyrazole-5-carbonitrile* (**5a**). Yield: 88%; m.p.: 240–242 °C; IR *ν*_max_ (cm^−1^): 3406 and 3157 (NH_2_, NH), 3022 (Ar–H), 2936 (C–H), 2208 (C≡N), 1598 (C=N), 1152 and 1215 (C–O–C); ^1^H-NMR δ ppm: 1.86 (s, 3H, –CH_3_), 4.51 (s, 1H, –CH–), 6.99–7.76 (m, 5H, aromatic ring) 8.45 (s, 2H, –NH_2_), and 12.02 (s, 1H, –NH); ^13^C-NMR δ ppm: 8.85, 34.69, 57.67, 96.48, 112.57, 119.69, 127.35, 134.73, 153.84, and 159.45; MS (ESI) *m*/*z* 252.10 (100.0%), 253.10 (16.6%), 254.11 (1.3%); Elemental analysis: calculated for C_14_H_12_N_4_O (C, H, N) 66.65, 4.79, 22.21, Found: 66.67, 4.75, 22.21.

*6-Amino-4-(4-chlorophenyl)-3-methyl-2,4-dihydropyrano[2,3-c]pyrazole-5-carbonitrile* (**5b**). Yield: 94%; m.p.: 228–230 °C; IR *ν*_max_ (cm^−1^): 3474 and 3220 (NH_2_ and NH)), 3050 (Ar–H), 2960 (C–H), 2208 (C≡N), 1598 (C=N), 1152 and 1215 (C–O–C), 744 (C–Cl); ^1^H-NMR δ ppm: 1.80 (s, 3H, –CH_3_), 4.58 (s, 1H, –CH–), 7.68–7.73 (m, 4H, Ar–H), 8.2 (s, 2H, –NH_2_), 12.06 (s, 1H, –NH); ^13^C-NMR δ ppm: 9.72, 38.18, 56.82, 98.0, 120.5, 129.5, 131.1, 132.3, 135.4, 144.2, 154.6, 160.8; MS (ESI) *m*/*z* 286.06 (100.0%), 288.06 (M + 2) (32.2%), 287.07 (15.3%); Elemental analysis: calculated for C_14_H_11_ClN_4_O (C, H, N, Cl) 58.65, 3.87, 19.54, 12.32, Found: 58.61, 3.82, 19.50, 12.30. 

*6-Amino-4-(4-fluorophenyl)-3-methyl-2,4-dihydropyrano[2,3-c]pyrazole-5-carbonitrile* (**5c**). Yield: 92%; m.p.: 172–174 °C; IR *ν*_max_ (cm^−1^): 3344 and 3290 (NH_2_, NH), 3280 (Ar–H), 2999 (C–H), 2222 (C≡N), 1640 (CN), 1260 (C–F), 1160 and 1220 (C–O–C); ^1^H-NMR δ ppm: 1.92 (s, 3H, –CH_3_), 4.6 (s, 1H, –CH–), 7.12–7.21 (m, 4H, Ar–H), 7.88 (s, 2H, –NH_2_), 12.04 (s, 1H, –NH); ^13^C-NMR δ ppm: 13.13, 25.5, 59.2, 113.4, 115.4, 115.8, 117.3, 130.6, 130.9, 132.2, 139.1, 159.9, 163.7, 176.1; MS (ESI) *m*/*z* 270.09 (100.0%), 271.10 (15.3%), 271.09 (1.5%); Elemental analysis: calculated for C_14_H_11_FN_4_O (C, H, N, F) 62.22, 4.10, 20.73, 7.03, Found: 62.22, 4.10, 20.73, 7.00.

*6-Amino-4-(4-methoxyphenyl)-3-methyl-2,4-dihydropyrano[2,3-c]pyrazole-5-carbonitrile* (**5d**). Yield: 94%; m.p.: 203–207 °C; IR *ν*_max_ (cm^−1^): 3483 and 3255 (NH_2_, NH), 3107 (Ar–H), 2930 (C–H), 2191 (C≡N), 1598 (C=N), 1450 (C–OCH_3_), 1152 and 1215 (C–O–C); ^1^H-NMR δ ppm: 1.79 (s, 3H, –CH_3_), 3.74 (s, 3H, –OCH_3_), 4.51 (s, 1H, –CH–), 6.80–7.0 (m, 4H, Ar–H), 8.2 (s, 2H, –NH_2_), 12.0 (s, 1H, –NH); ^13^C-NMR δ ppm: 9.5, 37.5, 55.4, 58.4, 98.1, 114.7, 121.2, 129.8, 145.5, 147.8, 155.3, 159.9, 163.0; MS (ESI) *m*/*z* 282.11 (100.0%), 283.12 (16.5%), 284.12 (1.7%); Elemental analysis: calculated for C_15_H_14_N_4_O_2_ (C, H, N) 63.82, 5.00, 19.85, Found: 63.78, 5.05, 19.82.

*6-Amino-4-(4-hydroxyphenyl)-3-methyl-2,4-dihydropyrano[2,3-c]pyrazole-5-carbonitrile* (**5e**). Yield: 88%; m.p.: 219 to 221 °C; IR *ν*_max_ (cm^−1^): 3500 (OH) 3455 and 3242 (NH_2_, NH), 3170 (Ar–H), 2940 (C–H), 2198 (C≡N), 1600 (C=N), 1145 and 1200 (C–O–C); ^1^H-NMR δ ppm: 2.00 (s, 3H, –CH_3_), 4.46 (s, 1H, –CH–), 5.44 (s 1H, –OH), 6.33–7.06 (m, 4H, Ar–H), 8.52 (s, 2H, –NH_2_), 11.90 (s, 1H, –NH); ^13^C-NMR δ ppm: 12.0, 25.0, 59.0, 113.6, 119.5, 121.0, 130.2, 141.5, 155.4, 163.5, 179.1, MS (ESI) *m*/*z* 268.10 (100.0%), 269.10 (15.4%), 270.10 (1.7%); Elemental analysis: calculated for C_14_H_12_N_4_O_2_ (C, H, N) 62.68, 4.51, 20.88, Found: 62.70, 4.48, 20.87.

*6-Amino-4-(4-hydroxy-3-methoxyhenyl)-3-methyl-2,4-dihydropyrano[2,3-c]pyrazole-5-carbonitrile* (**5f**). Yield: 85%; m.p.: 232 to 234 °C; IR *ν*_max_ (cm^−1^): 3500 (OH), 3333 and 3221 (NH_2_, NH), 3177 (Ar–H), 2830 (C–H), 2308 (C≡N), 1590 (C=N), 1440 (C–OCH_3_), 1200 and 1222 (C–O–C); ^1^H-NMR δ ppm: 2.00 (s, 3H, CH_3_), 3.80 (s, 3H, –OCH_3_), 4.46 (s, 1H, –CH–), 5.44 (s, 1H, –OH), 6.33–7.06 (m, 3H, Ar–H), 8.50 (s, 2H, –NH_2_), 11.95 (s, 1H, –NH); ^13^C-NMR δ ppm: 12.0, 25.0, 56.1, 59.0, 113.6, 115.8, 119.5, 127.0, 130.2, 141.5, 143.8, 147.4, 155.4, 163.5, 179.1; MS (ESI) *m*/*z* 298.11 (100.0%), 299.11 (16.5%), 300.11 (2.1%); Elemental analysis: calculated for C_15_H_14_N_4_O_3_ (C, H, N) 60.40, 4.73, 18.78, Found: 60.38, 4.70, 18.75.

*6-Amino-4-(3,4-dimethoxyphenyl)-3-methyl-2,4-dihydropyrano[2,3-c]pyrazole-5-carbonitrile* (**5g**). Yield: 87%; m.p.: 185 to 187 °C; IR *ν*_max_ (cm^−1^): 3332 and 3220 (NH_2_, NH), 3190 (Ar–H), 2850 (C–H), 2218 (C≡N), 1580 (C=N), 1400 (C–OCH_3_), 1190 and 1225 (C–O–C); ^1^H-NMR δ ppm: 2.07 (s, 3H, CH_3_), 3.70 (s, 6H, (–OCH_3_)_2_), 4.61 (s, 1H, –CH–), 6.80–7.16 (m, 3H, Ar–H), 7.52 (s, 2H, –NH_2_), 11.99 (s, 1H, –NH); ^13^C-NMR δ ppm: 11.5, 24.5, 55.4, 56.1, 70.4, 114.7, 115.2, 127.8, 129.2, 140.5, 143.8, 153.3; 159.9, 160.0, 176.1; MS (ESI) *m*/*z* 312.12 (100.0%), 313.13 (17.6%), 314.13 (2.1%); Elemental analysis: calculated for C_16_H_16_N_4_O_3_ (C, H, N) 61.53, 5.16, 17.94, Found: 61.50, 5.12, 17.92.

*6-Amino-3-methyl-4-(4-nitrophenyl)-2,4-dihydropyrano[2,3-c]pyrazole-5-carbonitrile* (**5h**). Yield: 88%; m.p.: 188–190 °C; IR *ν*_max_ (cm^−1^): 3351 and 3312 (NH_2_, NH), 3222 (Ar–H), 2936 (C–H), 2208 (C≡N), 1598 (C=N), 1345 (NO_2_), 1152 and 1215 (C–O–C); ^1^H-NMR δ ppm: 2.03 (s, 3H, CH_3_), 4.75 (s, 1H, –CH–), 7.54–8.65 (m, 4H, Ar–H), 8.45 (s, 2H, –NH_2_), 11.88 (s, 1H, –NH); ^13^C-NMR δ ppm: 11.9, 26.3, 71.4, 112.4, 121.3, 127.6, 133.1, 141.6, 144.9, 151.4, 159.0, 165.1; MS (ESI) *m*/*z* 297.09 (100.0%), 298.09 (15.4%), 299.09 (2.0%); Elemental analysis: calculated for C_14_H_11_N_5_O_3_ (C, H, N) 56.56, 3.73, 23.56, Found: 56.58, 3.70, 23.52.

*6-Amino-3-methyl-4-(thiophen-2-yl)-2,4-dihydropyrano[2,3-c]pyrazole-5-carbonitrile* (**5i**). Yield: 89%; m.p.: 222 to 224 °C; IR *ν*_max_ (cm^−1^): 3344 and 3289 (NH_2_, NH), 2191 (thiophene ring), 1647 (C≡N), 1600 (C=N), 1145–1014(–C–O–C–); ^1^H-NMR δ ppm: 1.92 (s, 3H, –CH_3_), 4.91 (s, 1H, –CH–), 6.40–7.45 (m, 3H, thiophene), 8.59 (s, 2H, –NH_2_), 12.11 (s, 1H, –NH); ^13^C-NMR δ ppm: 8.4, 30.2, 56.4, 95.9, 119.2, 123.1, 124.8, 139.5, 139.8, 148.0, 163.9, 177.2; MS (ESI) *m*/*z* 258.06 (100.0%), 259.06 (13.9%), 260.05 (4.5%); Elemental analysis: calculated for C_12_H_10_N_4_OS (C, H, N, S) 55.80, 3.90, 21.69, 12.41, Found: 55.82, 3.88, 21.72, 12.39.

*6-Amino-4-(4-(benzyloxy)phenyl)-3-methyl-2,4-dihydropyrano[2,3-c]pyrazole-5-carbonitrile* (**5j**). Yield: 88%; m.p.: 212 to 214 °C; IR *ν*_max_ (cm^−1^): 3333 and 3200 (NH_2_, NH), 3222 (Ar–H), 3055.25 (C–H), 2208 (C≡N), 1598 (C=N), 1152 and 1215 (C–O–C); ^1^H-NMR δ ppm: 1.93 (s, 3H, –CH_3_), 4.75 (s, 1H, –CH–), 5.14 (s, 2H, –CH_2_–), 6.38–7.47 (m, 9H, Ar–H), 8.12 (s, 2H, –NH_2_), 11.88 (s, 1H, –NH); ^13^C-NMR δ ppm: 13.3, 25.5, 59.2, 70.8, 113.4, 114.3, 117.3, 127.1, 128.9, 130.1, 136.7, 139.1, 156.0, 163.7, 176.8; MS (ESI) *m*/*z* 358.14 (100.0%), 359.15 (23.0%), 360.15 (2.9%); Elemental analysis: calculated for C_21_H_18_N_4_O_2_ (C, H, N) 70.38, 5.06, 15.63, Found: 70.40, 5.10, 15.70.

### 4.4. Evaluation of In Vitro Anticancer Activity

The in vitro anticancer activity [82] of the newly synthesized compounds in four concentrations was carried out by the Sulforhodamine B (SRB) assay against four human cancer cell lines viz., melanoma (SK-MEL-2), breast (MDA-MB-231), leukemia (K-562) and cervix (HeLa). The cells were maintained in RPMI 1640 medium supplemented with 10% fetal bovine serum and 2 mM L-glutamine. Briefly, 5 × 10^3^ cells/well were inoculated into 96-well microtiter plates and incubated at 37 °C in a CO_2_ incubator for 24 h. The next day cells were exposed to different concentrations of test samples and incubated under standard conditions for 48 h. After incubation, cells were fixed by the gentle addition of 50 µL of cold 30% (*w*/*v*) trichloroacetic acid (TCA) and incubated for 60 min at 4 °C. The supernatant was decanted and plates were washed gently under the tap water and air dried at room temperature. 50 µL of 0.4% (*w*/*v*) Sulforhodamine B (SRB) solution in 1% acetic acid was added to each of the wells, and plates were incubated for 20 min at room temperature. After staining, unbound dye was recovered and the residual dye was removed by washing with 1% acetic acid. The plates were air dried. Bound stain was subsequently eluted with 100 µL of 10 mM trizma base, and the absorbance was read on an ELISA plate reader (Model Sunrise, Tecan, Seestrasse 103, Mannedorf, Switzerland) at a wavelength of 540 nm with 690 nm reference wavelength. The optical density of treated cells were compared with that of the control cells and growth inhibition was calculated as a percent value.

### 4.5. Molecular Docking Study

The molecular docking study was initiated with the sketching of the 2D form of the structures of all synthesized compounds using the sketch modules of SYBYL-X 2.1.1 (Certara. L.P., Princeton, NJ, USA) The 2D forms of the compounds were then subjected to the ligand library preparation module by using the Surface preparation protocol for searching which generates single lowest strain energy tautomer/stereoisomers and all necessary structural properties were added and the final prepared conformations are stored in SYBYL-Mol2 file format. To perform molecular docking a three dimensional X-ray crystal structure of tubulin (**PDB ID: 1SA0** Resolution 3.58 Å) complex with colchicine and a stathmin-like domain was used. The selected target protein was subjected to the prepare protein structure module from the Biopolymer menu were they analyzed for termini treatment, hydrogen addition, protonation, type, atoms type, charge addition, and side chain fixation. The target protein was subjected to staged minimization by keeping the default parameters and selecting the Tripos force field. The co-crystallized ligand molecules were extracted from the target protein and saved as reference ligand to define molecular docking similarly with other substructures and water molecules are removed and the final prepared target protein structures was stored in Mol2 file format. Molecular docking was defined after the target protein structure ligand library are prepared. The Surflex-Dock module of SYBYL 2.1.1 molecular docking was used to define ligand docking. The docking mode was selected as a Surflex-Dock control file (.sfxc) which contains information about the protein and the method used to generate the protomol. The protomol is constructed using the co-crystallized ligand as a reference. The prepared ligand library was used as source of ligands and Surflex docking input options are set as the defaults with a search density of 6.00, number of spin alignments at 12 and output options maximum number of poses per ligand as of 1 and minimum RSMD between final poses at 0.50 Å. Nice level was set to 0 and the number of processors was used for molecular docking was 2 and the molecular docking result was saved in the docking directory.

### 4.6. In Silico ADMET Prediction

A computational study of the synthesized compounds **5a**–**j** was performed for prediction of ADMET properties. The absorption, distribution, metabolism, excretion and toxicity (ADMET) properties of all the compounds were predicted using the ADMET predictor FAFDrugs2 (Lagorce et al. Paris, France) which runs on the Linux OS. In the present study, we have calculated the molecular volume (MV), molecular weight (MW), predicted octanol-water partition coefficient (log Po/w), number of hydrogen bond acceptors (n-ON), number of hydrogen bonds donors (*n*-OHNH), percentage of human oral absorption (% ABS), Van der Waals surface area of polar nitrogen and oxygen atoms (polar surface area), and LogS (water solubility).

## 5. Conclusions

In the current study, we have developed an easy, efficient, and green synthetic protocol to prepare 6-amino-4-(substituted phenyl)-3-methyl-2,4-dihydropyrano[2,3-*c*]pyrazole-5-carbonitriles **5a**–**j** by one pot condensations of various aromatic aldehydes, propanedinitrile, hydrazine hydrate and ethyl acetoacetate and using the ionic liquid triethylammonium hydrogen sulphate ([Et_3_NH][HSO_4_]) as a green reaction medium and also as the catalyst. This method overcomes the disadvantages associated with conventional refluxing which requires many hours for completion of the reaction and also avoids the use of harmful solvents and reagents for synthesis. The remarkable benefits of this synthetic strategy are as follows: (1) reactions were carried at room temperature and require much less time for completion hence this methodology saves time and electricity, (2) the use of a non-toxic and economically feasible catalyst which avoids the use of conventional hazardous solvents, (3) and shortened work-up procedure. 

Moreover, the synthesized compounds exhibited excellent to good activity against melanoma, breast and leukemia cancer cell lines. Our computational molecular docking study demonstrated that **5b** and **5d** are the most active amongst the synthesized derivatives and have the potential for cytotoxic action towards cancer cells, supporting the experimental anticancer activity results. Compound **5b** showed excellent in vitro anticancer activity against a melanoma (SK-MEL-2) and breast cancer cell line (MDA-MB-231) with concentrations of <0.1 and 0.74 µM, respectively, also proved by the docking score of 6.4039 which shows that it inhibits enzyme tubulin α/β. Compound **5d** showed excellent in vitro anticancer activity against a melanoma (SK-MEL-2) cell line at a concentration <0.1 µM and similarly, it also inhibits the enzyme tubulin α/β with a docking score of 5.4734. In conclusion, compounds **5b** and **5d** not only give good docking scores, but also show growth inhibition concentration 50% (GI_50_) anticancer activity even at a very low concentration <0.1 µM. As compared to the standard drug ADR, these derivatives were found to be the competent moieties with potent anticancer activity. Predictions of pharmacokinetic parameters suggest that the synthesized compounds have high oral drug bioavailability potential. Thus, the results of the molecular docking study and anticancer activity data of these 6-amino-4-(substituted phenyl)-3-methyl-2,4-dihydro-pyrano[2,3-*c*]pyrazole-5-carbonitriles proves that these compounds have potential to be developed as lead anticancer molecules and can act as an excellent scaffold for lead optimization and drug discovery.

## Figures and Tables

**Table 1:** Selection of reaction media for the synthesis of compound **5b**.

Solvent	Temperature (°C)	Time (min)	Yield (%)
Polyethylene glycol (PEG)	80	60	72
Deep eutectic solvent of cholinechloride:urea	80	20	92
Ionic liquid (*N*-methylpyridiniumtosylate)	120	75	62
Ionic liquid (triethylammonium hydrogen sulphate[Et_3_NH][HSO_4_])	R.T.	15	94

R.T. = Room temperature.

**Table 2:** Effect of ionic liquid concentration on reaction time and yield of **5b**.

[Et_3_NH][HSO_4_] mol %	Time (min)	Yield (%)
-	60	00
5	50	50
10	45	65
15	15	70
20	15	94
25	15	85

**Table 3:** Reusability of the ionic liquid in the model reaction synthesis of **5b**.

Run	Time (min)	Yield (%)
1	15	94
2	15	82
3	15	78
4	15	75

**Table 4:** In vitro anticancer activity and molecular docking of selected synthesized compounds.

Compounds	GI_50_ Against Cancer Cell Line in µM				Total Score -Logki	Crash Score	Polar Score
	MAD-MB-231	HeLa	SK-MEL-2	K-562			
**5b**	0.74	>100	<0.1	11.2	6.4039	−0.9801	2.0598
**5d**	25.76	>100	<0.1	6.41	5.4734	−1.3871	4.449
**5g**	2.02	>100	2.92	40.73	4.6919	−0.4209	5.4948
**5h**	49.84	>100	0.12	4.57	4.5247	−0.184	3.7291
**5j**	66.65	>100	<0.1	9.47	4.6555	−0.7369	3.5549
**ADR**	<0.1	0.03	<0.1	<0.1	NA	NA	NA
**CL2**	NA	NA	NA	NA	5.1969	−0.8748	3.5552

CL2: Co-crystallized ligand and Adriamycin (ADR), the standard drug used for the anticancer activity assays.

**Table 5:** In silico physicochemical (drug-likeliness) properties of the synthesized compounds **5a**–**j** with reference ligand CL2.

Lig_ID	%ABS (<100%)	MW (<500)	LogP (<5)	PSA (<150)	*n*-RotB (<10)	*n*-RigB (<25)	HBD (<5)	HBA (<10)	RatioH/C (<1)	Toxicity
**5a**	78.64	252.3	2.64	87.72	1	17	2	2	0.357	Non Toxic
**5b**	78.64	286.7	3.29	87.72	1	17	2	2	0.428	Non Toxic
**5c**	78.64	270.3	2.78	87.72	1	17	2	2	0.428	Non Toxic
**5d**	75.55	282.3	2.65	96.95	2	17	2	3	0.4	Non Toxic
**5e**	71.76	268.3	2.34	108	1	17	3	3	0.428	Non Toxic
**5f**	68.57	298.3	2.35	117.2	2	17	3	4	0.466	Non Toxic
**5g**	72.37	312.3	2.65	106.2	3	17	2	4	0.437	Non Toxic
**5h**	62.93	297.3	3.07	127.7	2	18	2	4	0.571	Non Toxic
**5i**	78.64	258.3	2.7	116	1	16	2	3	0.5	Non Toxic
**5j**	75.55	358.4	4.22	96.95	4	23	2	3	0.5	Non Toxic
**CL2**	75	252.3	2.64	87.72	1	17	2	2	0.357	Non Toxic

% ABS: Percent absorption, MW: molecular weight, LogP: logarithm of partition coefficient of compound between *n*-octanol and water, PSA: Polar surface area, n-Rot Bond: number of rotatable bonds, n-Rig Bond: number of rigid bonds, HBA: hydrogen bond acceptors and HBD: hydrogen bond donor.

## Supplementary Materials

The following are available online, S1: Spectral data, S2: images, S3: Graphs, S4: Table, S5: Compound structures with names.
