# Supplementary material for: Ionic Liquid-Catalyzed Green Protocol for Multi-Component Synthesis of Dihydropyrano[2,3-c]pyrazoles as Potential Anticancer Scaffolds
Source: Molecules. 2017 Sep 28;22(10):1628. doi: 10.3390/molecules22101628 (PMC6151819; doi:10.3390/molecules22101628)
Supplement: Supplementary file 1 [file molecules-22-01628-s001.docx]

**Supplementary Data S1**

**Mass Spectra: 5b) 6-amino-4-(4-chlorophenyl)-3-methyl-2,4-dihydropyrano[2,3-c]pyrazole-5-carbonitrile**


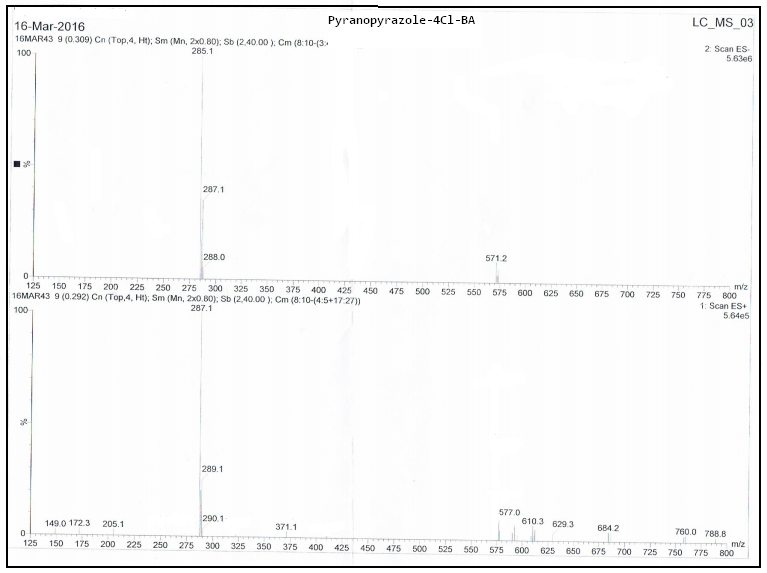


**^1^H NMR Spectra: 5b) 6-amino-4-(4-chlorophenyl)-3-methyl-2,4-dihydropyrano[2,3-c]pyrazole-5-carbonitrile**

**^^**

**^13^C NMR Spectra: 5b) 6-amino-4-(4-chlorophenyl)-3-methyl-2,4-dihydropyrano[2,3-c]pyrazole-5-carbonitrile**

DMSO_d6_

CDCl_3_

**Mass Spectra: 5d) 6-amino-4-(4-methoxyphenyl)-3-methyl-2,4-dihydropyrano[2,3-c]pyrazole-5-carbonitrile**


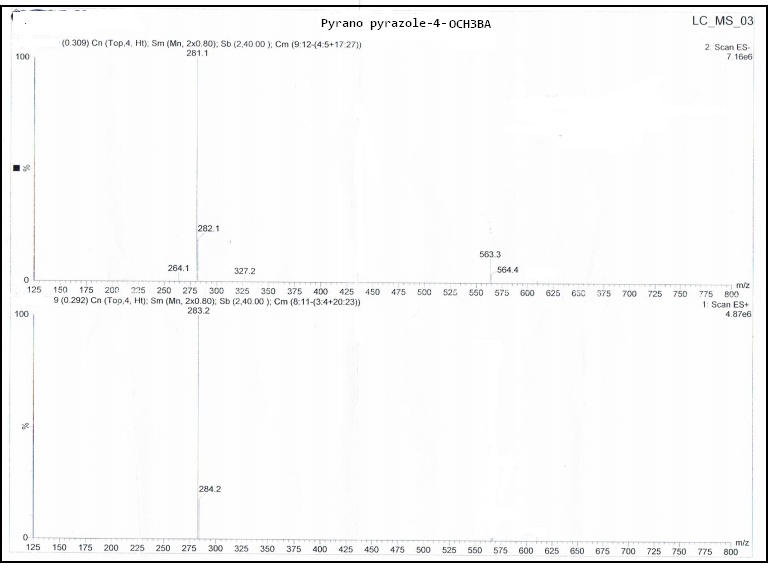


**^1^H NMR Spectra:5d) 6-amino-4-(4-methoxyphenyl)-3-methyl-2,4-dihydropyrano[2,3-c]pyrazole-5-carbonitrile**

**^13^C NMR Spectra: 5d) 6-amino-4-(4-methoxyphenyl)-3-methyl-2,4-dihydropyrano[2,3-c]pyrazole-5-carbonitrile**

DMSO_d6_

CDCl_3_

**Supplementary Data S2: *In Vitro* Anticancer Activity Images**

**Fig.1:** Images for anticancer activity against (MDA-MB-235) Breast Cancer and (SK-MEL-2) Melanoma cancer Cell line.


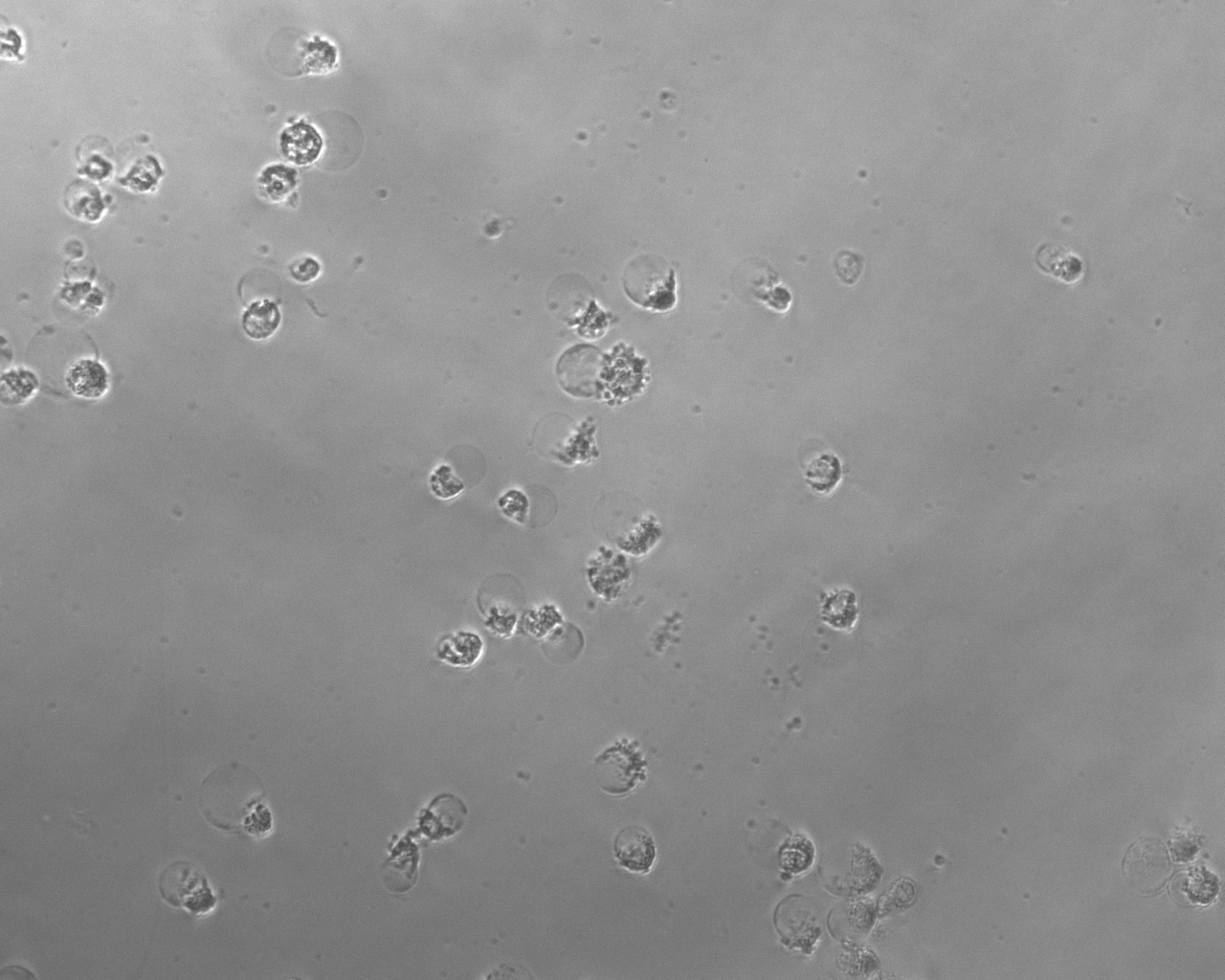


MDA-MB-235-positive control


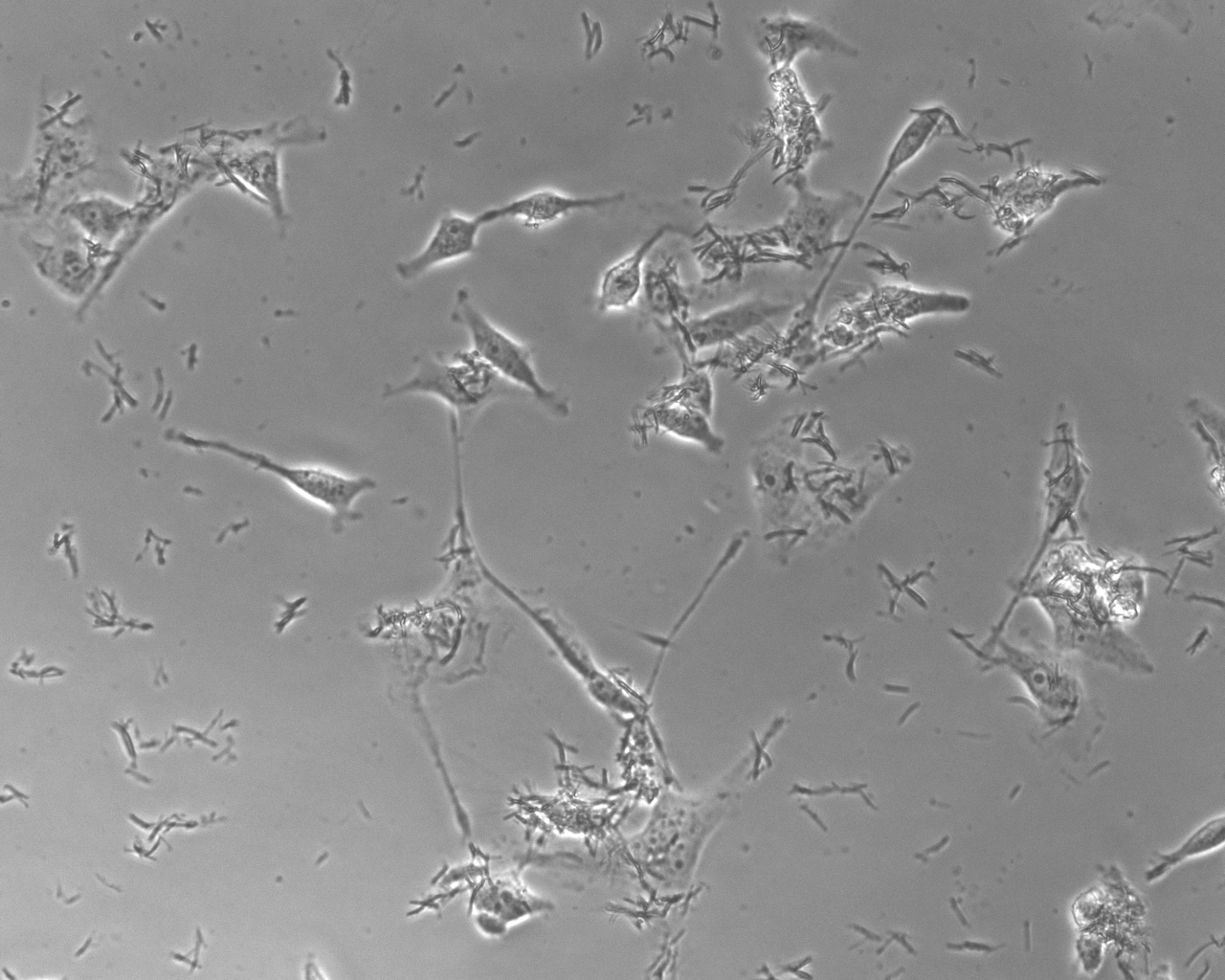


MDA-MB-235( 5b)


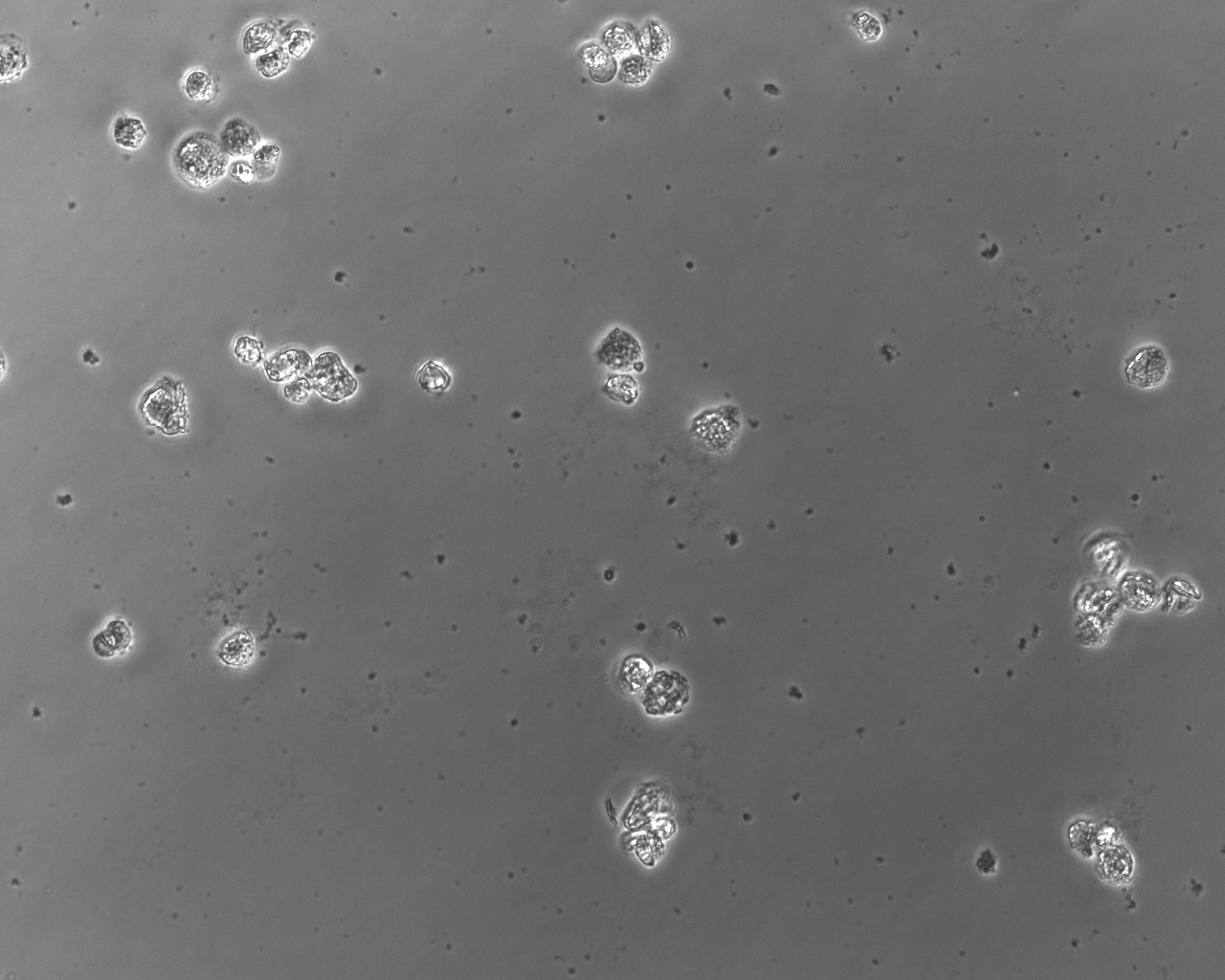


SK-MEL-2 positive control


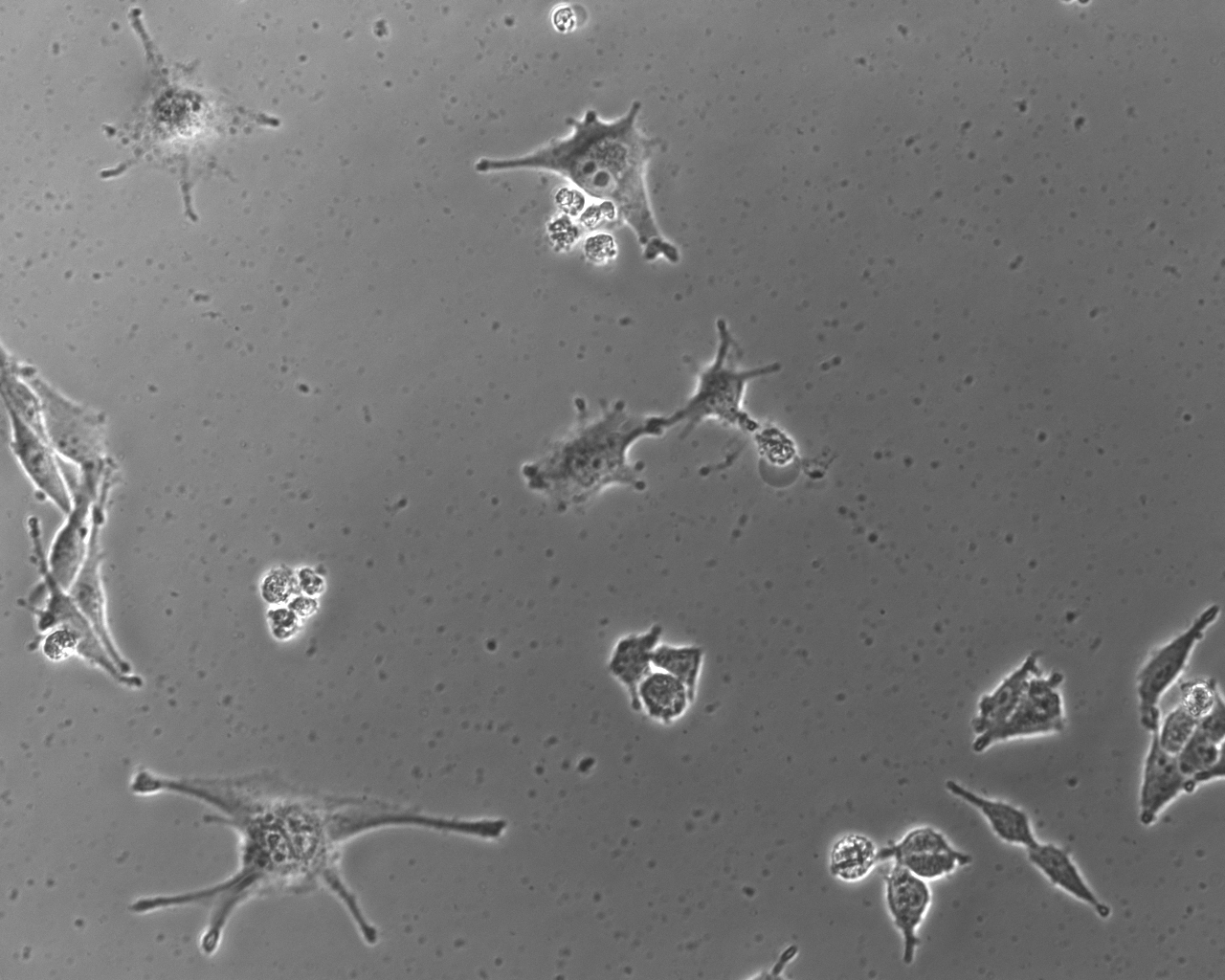


SK-MEL-2 (5b)


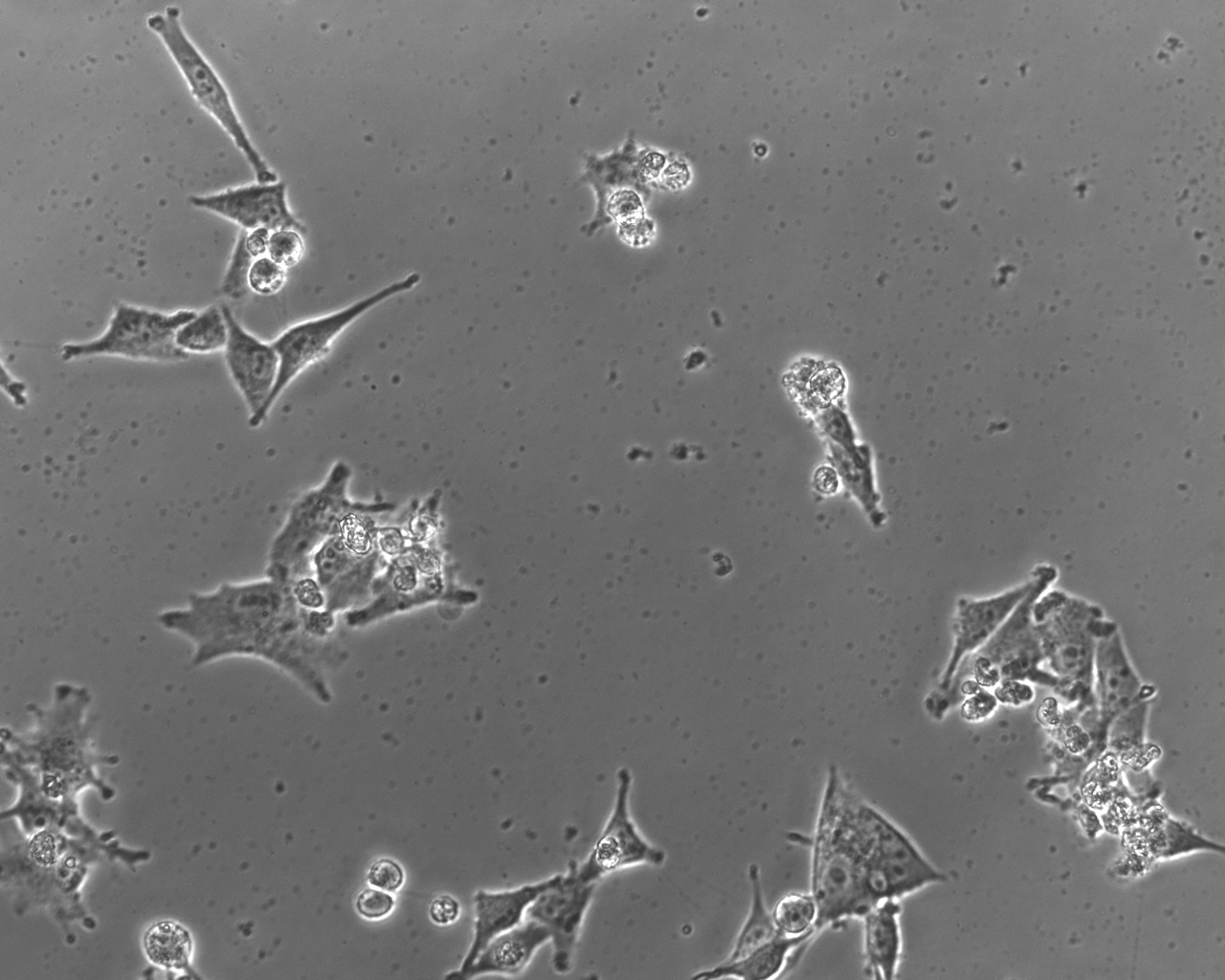


SK-MEL-2 (5d)


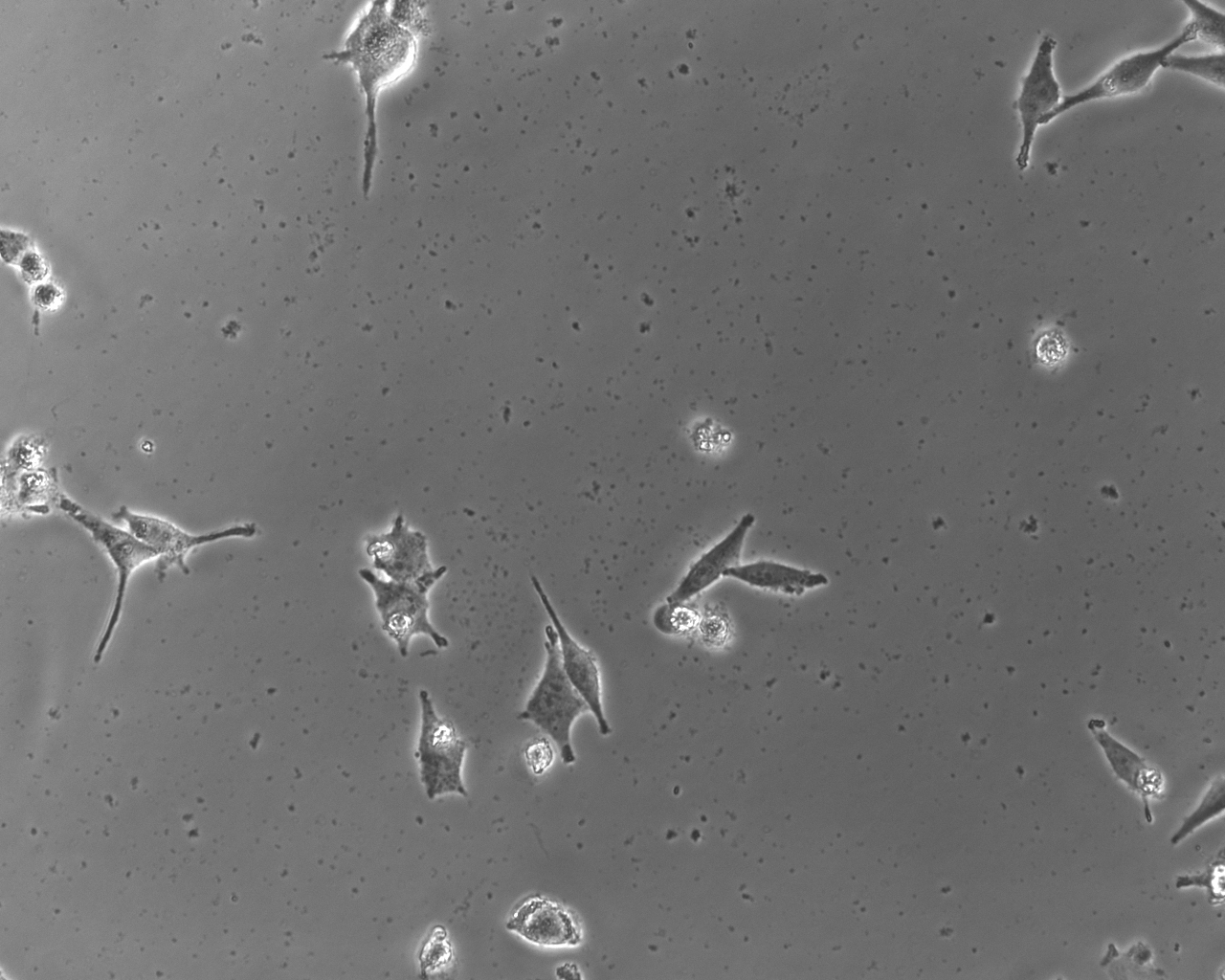


SK-MEL-2 (5g)


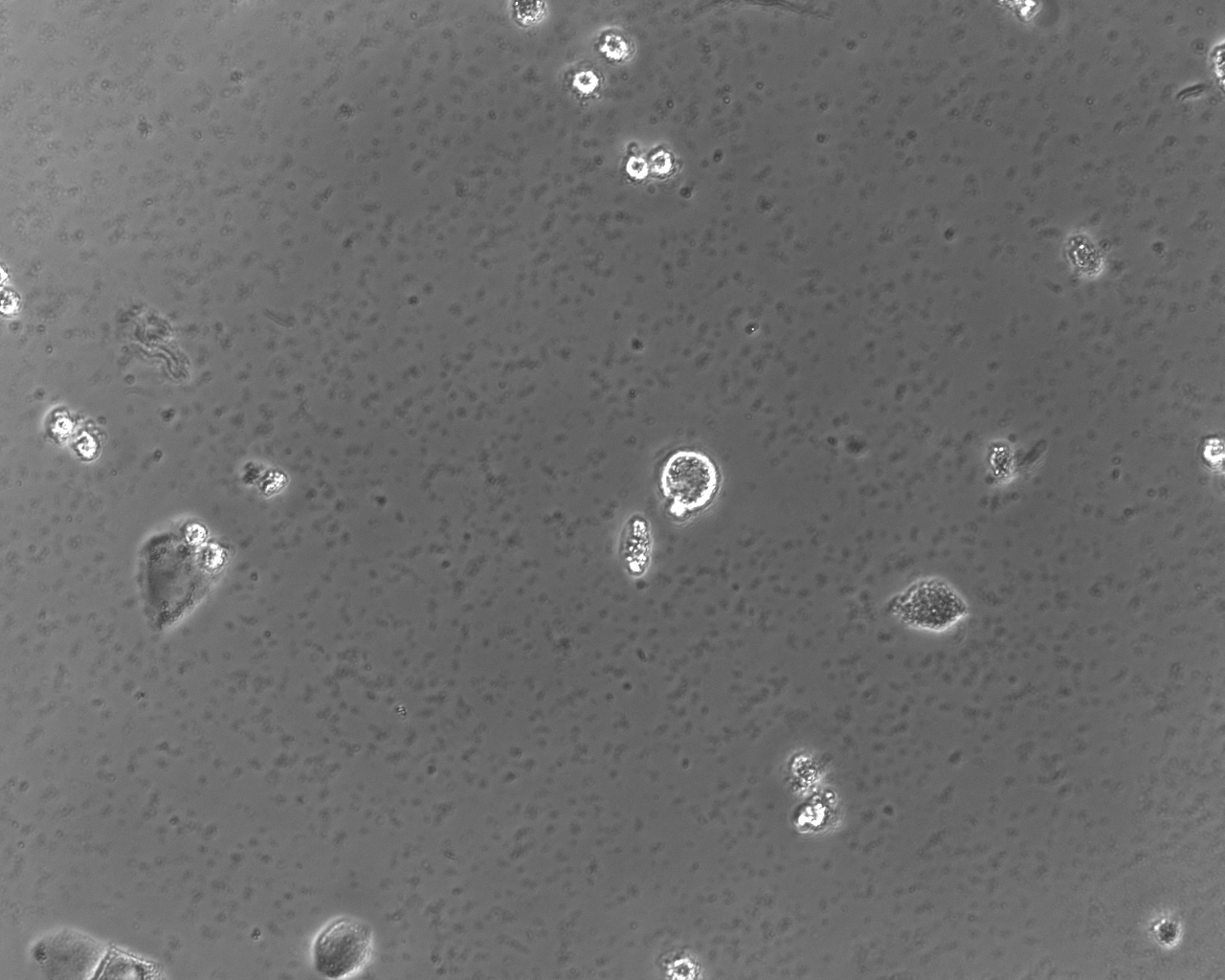


SK-MEL-2 (5h)


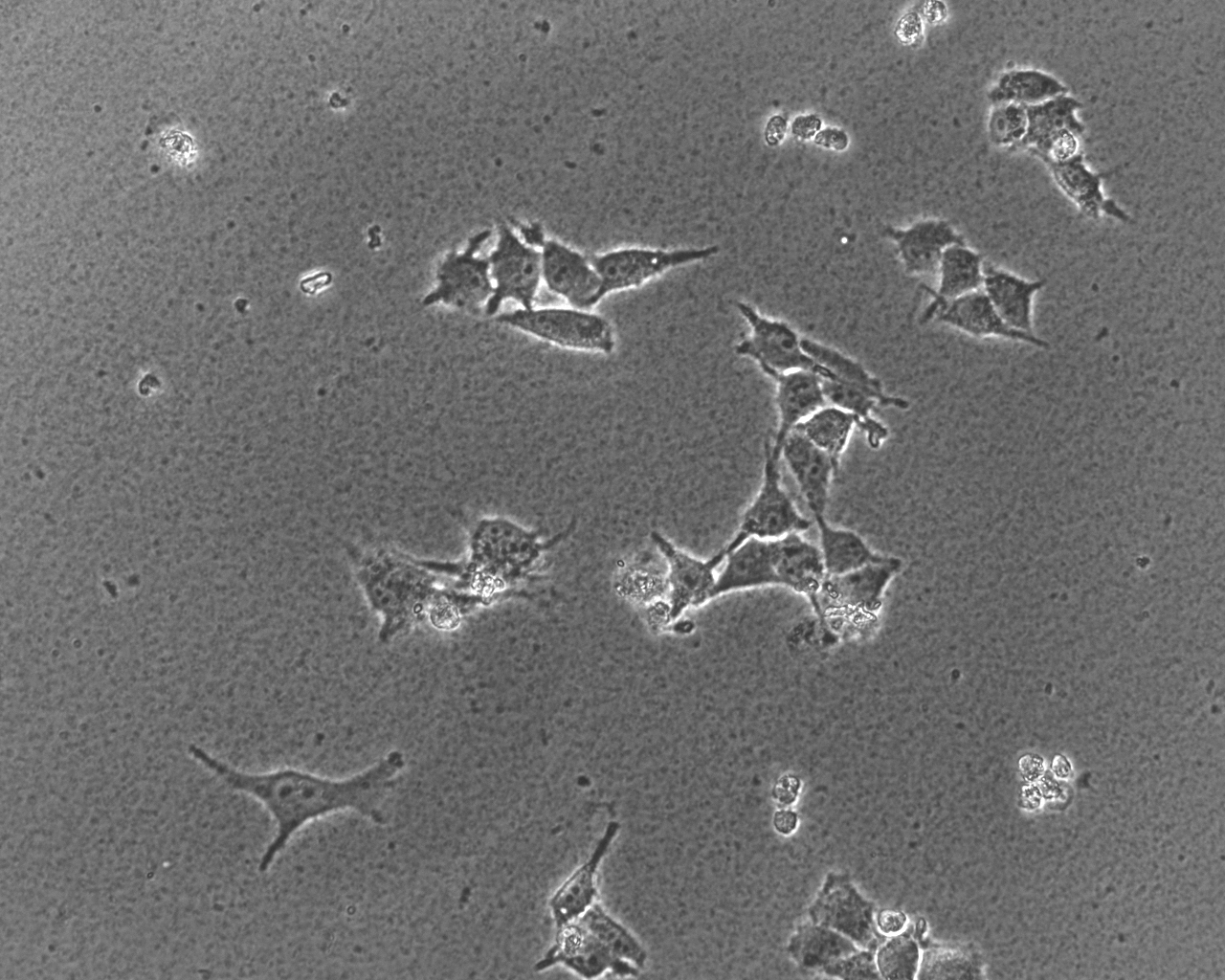


SK-MEL-2 (5j)

**Supplementary Data S3: Graphs**

**Fig.2**. Graphical Representation of Growth Inhibition Curve of Breast Cancer Cell Line, Melanoma Cancer Cell Line, Leukemia Cancer Cell Line and Cervical Cancer Cell Line


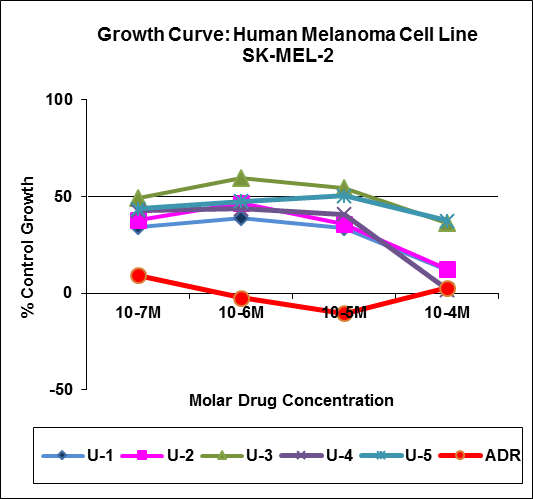


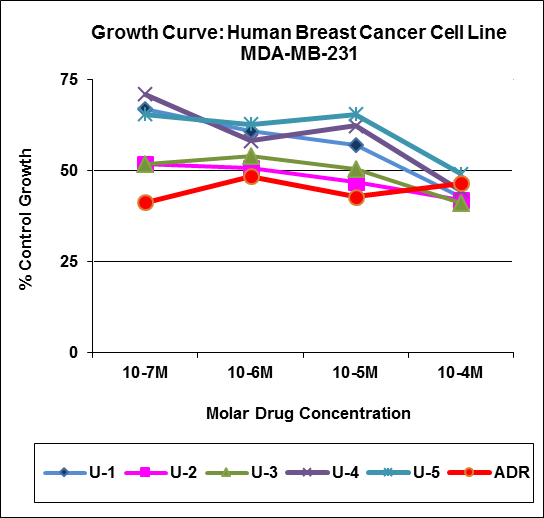


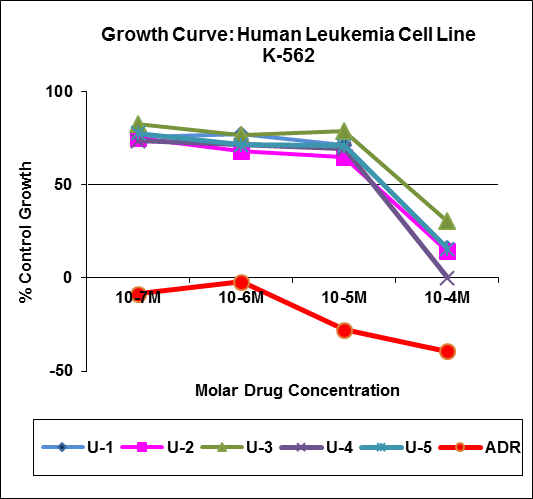


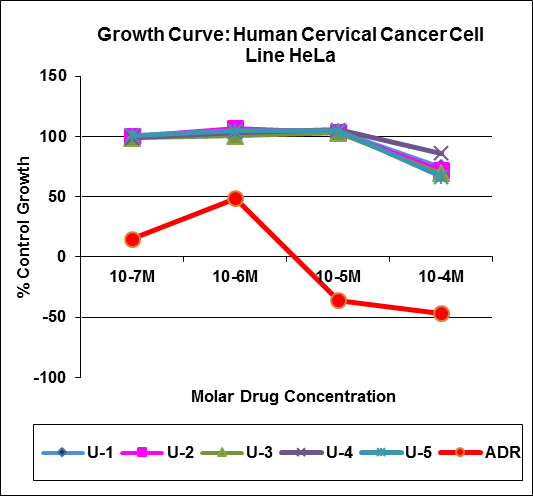


| **Table S4.** Physical characterization of 6-amino-4-(substituted phenyl)-3-methyl-2,4-dihydropyrano [2,3-c]pyrazole-5-carbonitrile 5a-5j | | | | | |
| --- | --- | --- | --- | --- | --- |
| **Compound**  **code** | **Ar** | **Mol. Wt.** | **Yield (%)** | **M.P. (˚C)** | **Analysis (%) Found and [calculated]**  **C H N** |
| 5a |  | 252.10 | 88 | 240-242 | C,66.65; H, 4.79; N,22.21  [C,66.67; H, 4.75; N, 22.21] |
| 5b |  | 286.06 | 94 | 228-230 | C,58.65; H, 3.87; N, 19.54  [C,58.61; H, 3.82; N, 19.50] |
| 5c |  | 270.09 | 92 | 172-174 | C,62.22; H, 4.10; N, 20.73  [C, 62.22; H, 4.10; N, 20.73] |
| 5d |  | 282.11 | 94 | 205-207 | C,63.82; H, 5.00; N, 19.85  [C, 63.78; H, 5.05; N, 19.82] |
| 5e |  | 268.10 | 88 | 219-221 | C,62.68; H, 4.51; N, 20.88  [C,62.70; H, 4.48; N, 20.87] |
| 5f |  | 298.11 | 85 | 232-234 | C,60.40; H, 4.73; N, 18.78  [C,60.38; H, 4.70; N, 18.75] |
| 5g |  | 312.12 | 87 | 185-187 | C,61.53; H, 5.16; N, 17.94  [C,61.50; H, 5.12; N, 17.92] |
| 5h |  | 297.09 | 88 | 188-190 | C,56.56; H, 3.73; N, 23.56  [C,56.58; H, 3.70; N, 23.52] |
| 5i |  | 258.06 | 89 | 222-224 | C,55.80; H, 3.90; N, 21.69  [C,55.82; H, 3.88; N, 21.72] |
| 5j |  | 358.14 | 88 | 212-214 | C,70.38; H, 5.06; N, 15.63  [C,70.40; H, 5.10; N, 15.70] |

Reference for comparison of melting point or reaction yields:

Bhosle, M.R.; Khillare,L.D. Dhumal,S.T.; Mane,R.A. A facile synthesis of 6-amino-2H, 4H-pyrano[2,3-F]pyrazole-5- 4 carbonitriles in deep eutectic solvent. *Chinese Chemical Letters,***2015**,3497,1-5.

**S5: Structure and name of synthesised derivatives**
